# Supplementary material for: County-Wide Mortality Assessments Attributable to PM2.5 Emissions from Coal Consumption in Taiwan
Source: Int J Environ Res Public Health. 2022 Jan 30;19(3):1599. doi: 10.3390/ijerph19031599 (PMC8835574; doi:10.3390/ijerph19031599)
Supplement: Supplementary file 1 [file ijerph-19-01599-s001.zip › Table S1.pdf]

Table S1. Disease-specific population attributable fraction for adult death and pre-mature death due to $PM_{2.5}$ in different cities/counties, Taiwan

City/County	IHD	Stroke	LC	COPD	Overall PAF
Taipei City	14.0% (20.7%)*	21.8% (34.0%)	13.9% (13.9%)	11.4% (11.3%)	16.1% (21.4%)
Taichung City	18.5% (26.3%)	29.0% (41.9%)	17.9% (17.8%)	14.6% (14.5%)	21.1% (27.0%)
Tainan City	19.8% (29.1%)	31.1% (44.5%)	19.6% (19.6%)	16.0% (15.6%)	22.9% (29.7%)
Kaohsiung City	22.6% (31.9%)	35.6% (49.2%)	22.2% (22.2%)	17.9% (17.9%)	25.8% (32.1%)
Keelung City	12.3% (17.6%)	20.5% (30.4%)	11.8% (11.7%)	10.0% (9.9%)	14.6% (19.1%)
Hsinchu City	16.3% (24.2%)	25.2% (37.3%)	16.0% (16.0%)	12.9% (13.2%)	18.9% (24.9%)
Chiayi City	22.7% (31.5%)	33.6% (49.5%)	22.3% (22.3%)	18.6% (18.3%)	25.3% (31.3%)
New Taipei City	14.6% (20.8%)	23.5% (34.3%)	13.9% (13.9%)	11.2% (11.5%)	16.7% (21.3%)
Taoyuan City	15.0% (21.6%)	23.7% (35.2%)	14.6% (14.6%)	12.1% (12.1%)	17.7% (23.5%)
Hsinchu County	15.1% (22.9%)	24.2% (36.5%)	14.5% (14.6%)	11.8% (12.0%)	18.3% (26.9%)
Ilan County	10.6% (16.5%)	17.7% (26.9%)	10.6% (10.5%)	8.7% (8.6%)	12.7% (17.9%)
Miaoli County	14.7% (22.2%)	23.2% (35.5%)	14.8% (14.9%)	12.6% (12.1%)	17.5% (23.9%)
Changhua County	18.0% (26.7%)	28.9% (42.0%)	18.2% (18.2%)	14.8% (15.0%)	20.8% (28.0%)
Nantou County	19.3% (27.1%)	30.1% (42.3%)	18.9% (18.8%)	15.4% (15.4%)	21.9% (28.7%)
Yunlin County	18.2% (26.9%)	29.3% (42.2%)	18.2% (18.2%)	14.6% (14.7%)	21.0% (27.7%)
Chiayi County	18.8% (28.1%)	28.8% (44.3%)	19.0% (19.1%)	15.6% (15.8%)	21.4% (27.1%)
Pingtung County	17.7% (25.6%)	28.1% (40.4%)	17.2% (17.1%)	13.6% (14.0%)	20.6% (26.8%)
Hualian County	11.1% (16.7%)	19.5% (28.3%)	10.9% (10.9%)	9.0% (8.9%)	14.1% (19.5%)
Taitung County	9.3% (12.3%)	13.2% (20.1%)	7.5% (7.5%)	6.2% (6.4%)	9.6% (12.9%)
Taiwan	16.9% (24.2%)	26.7% (39.0%)	16.7% (16.8%)	13.7% (14.1%)	19.6% (25.5%)

Abbreviation: PAF: population attributable fraction, IHD: Ischemic Heart Disease, LC: Lung Cancer, COPD: Chronic Obstruct Pulmonary Disease

*Data is shown as disease-specific PAF estimates for adult death (pre-mature death); Unit in percentage.
