# Supplementary material for: County-Wide Mortality Assessments Attributable to PM2.5 Emissions from Coal Consumption in Taiwan
Source: Int J Environ Res Public Health. 2022 Jan 30;19(3):1599. doi: 10.3390/ijerph19031599 (PMC8835574; doi:10.3390/ijerph19031599)
Supplement: Supplementary file 1 [file ijerph-19-01599-s001.zip › Table S2.pdf]

Table S2. Disease-specific total deaths attributable to ambient $PM_{2.5}$ in different cities/counties, Taiwan

	IHD	Stroke	LC	COPD	Total Death
City/County	n (95%CI)	n (95%CI)	n (95%CI)	n (95%CI)	n (95%CI)
Taipei City	144.8 (116.2~171.4)	263.3 (224.9~302.0)	137.6 (119.3~155.8)	60.5 (15.6~117.7)	606.2 (529.7~688.4)
Taichung City	143.6 (119.5~164.7)	312.2 (270.8~350.9)	155.5 (138.2~171.2)	81.1 (22.3~150.7)	692.3 (612.3~778.3)
Tainan City	138.1 (116.8~159.0)	310.7 (270.3~349.5)	152.3 (135.1~168.4)	83.1 (23.2~151.7)	684.2 (600.4~768.6)
Kaohsiung City	251.4 (214.1~288.4)	446.3 (395.1~492.9)	235.4 (211.0~257.5)	113.3 (41.0~194.1)	1046.3 (944.6~1155.9)
Keelung City	21.0 (17.5~24.2)	41.3 (36.1~46.4)	17.4 (15.6~19.3)	8.4 (2.8~14.8)	88.1 (79.0~97.3)
Hsinchu City	28.0 (23.1~32.2)	47.0 (40.5~53.4)	21.7 (19.3~24.1)	7.4 (1.6~14.5)	104.1 (93.6~114.6)
Chiayi City	18.9 (15.7~21.7)	38.6 (33.1~43.7)	18.3 (16.3~20.0)	13.0 (4.1~22.1)	88.7 (78.0~100.4)
New Taipei City	181.2 (154.4~209.0)	337.7 (291.2~379.0)	183.9 (164.9~204.7)	73.7 (25.8~141.3)	776.4 (695.7~869.5)
Taoyuan City	80.2 (65.8~94.1)	219.0 (188.3~249.2)	95.2 (84.1~106.5)	42.7 (10.8~82.2)	437.1 (384.3~492.6)
Hsinchu County	32.6 (26.8~38.0)	76.7 (66.0~87.5)	19.8 (17.2~22.4)	12.2 (3.2~23.6)	141.3 (125.8~159.5)
Ilan County	24.3 (20.3~28.2)	55.1 (48.0~61.9)	24.3 (21.3~27.1)	12.2 (3.7~23.5)	116.0 (102.4~130.6)
Miaoli County	41.4 (34.1~48.3)	96.2 (83.0~109.9)	31.1 (27.8~34.8)	25.1 (6.7~47.5)	193.7 (168.2~220.8)
Changhua County	99.7 (81.2~116.5)	183.1 (158.9~206.3)	98.3 (86.6~110.7)	54.5 (16.8~97.7)	435.6 (383.3~490.3)
Nantou County	44.4 (37.2~51.1)	97.4 (84.9~109.6)	41.5 (36.8~46.1)	33.8 (10.3~60.6)	217.1 (186.8~247.2)
Yunlin County	72.0 (59.8~84.4)	135.2 (116.6~153.1)	76.4 (65.9~87.1)	33.9 (10.4~60.1)	317.4 (279.9~357.1)
Chiayi County	53.2 (44.2~61.7)	106.5 (91.2~121.2)	60.7 (53.9~67.6)	32.1 (10.2~56.8)	252.4 (221.6~284.2)
Pingtung County	76.1 (58.5~95.6)	160.1 (129.0~190.3)	60.6 (48.1~74.2)	36.9 (13.7~65.0)	333.7 (265.2~400.4)
Hualian County	16.0 (13.1~18.9)	51.6 (45.0~58.0)	17.5 (15.4~19.5)	8.6 (2.3~15.6)	93.7 (83.7~104.2)
Taitung County	17.0 (14.4~19.6)	19.0 (16.6~21.3)	8.2 (7.2~9.3)	4.4 (1.2~8.5)	48.6 (43.4~54.0)
Taiwan	1483.8 (1413.7~1558.8)	2996.8 (2886.8~3108.5)	1455.6 (1404.3~1504.2)	736.7 (564.6~908.7)	6672.9 (6441.9~6921.6)

Abbreviation: IHD: Ischemic Heart Disease, LC: Lung Cancer, COPD: Chronic Obstruct Pulmonary Disease, CI: Confidence Interval; Unit in deaths/year.
