# Supplementary material for: County-Wide Mortality Assessments Attributable to PM2.5 Emissions from Coal Consumption in Taiwan
Source: Int J Environ Res Public Health. 2022 Jan 30;19(3):1599. doi: 10.3390/ijerph19031599 (PMC8835574; doi:10.3390/ijerph19031599)
Supplement: Supplementary file 1 [file ijerph-19-01599-s001.zip › Table S3.pdf]

Table S3. Disease-specific deaths attributable to $PM_{2.5}$ from all coal-fired plants in different cities/counties, Taiwan

	IHD	Stroke	LC	COPD	Total Death
City/County	n (95%CI)	n (95%CI)	n (95%CI)	n (95%CI)	n (95%CI)
Taipei City	4.4 (3.4~5.5)	7.6 (6.0~9.2)	4.2 (3.5~5.0)	1.8 (0.4~3.5)	18.1 (14.5~21.7)
Taichung City	7.0 (5.6~8.6)	14.1 (11.3~16.6)	7.7 (6.4~9.2)	3.9 (0.9~6.9)	32.7 (26.6~38.8)
Tainan City	8.2 (6.7~9.8)	16.9 (14.1~19.5)	9.2 (7.8~10.6)	4.9 (1.5~8.3)	39.1 (33.1~45.4)
Kaohsiung City	14.5 (12.3~16.7)	23.0 (20.5~25.5)	13.8 (12.4~15.3)	6.5 (2.4~10.6)	57.8 (51.6~63.7)
Keelung City	1.0 (0.7~1.2)	1.8 (1.4~2.2)	0.8 (0.6~1.0)	0.4 (0.1~0.7)	3.9 (3.1~4.8)
Hsinchu City	1.2 (0.9~1.5)	1.8 (1.4~2.3)	0.9 (0.7~1.1)	0.3 (0.1~0.6)	4.2 (3.3~5.2)
Chiayi City	1.0 (0.8~1.3)	1.9 (1.5~2.4)	1.0 (0.8~1.2)	0.7 (0.2~1.2)	4.6 (3.6~5.7)
New Taipei City	7.9 (6.3~9.7)	13.8 (11.3~16.6)	8.2 (6.7~9.7)	3.3 (1.1~5.9)	33.2 (27.4~39.7)
Taoyuan City	4.3 (3.3~5.2)	11.0 (9.0~13.3)	5.2 (4.2~6.2)	2.3 (0.6~4.3)	22.7 (18.5~27.6)
Hsinchu County	1.4 (1.2~1.7)	3.1 (2.6~3.6)	0.9 (0.7~1.0)	0.5 (0.1~1.0)	5.9 (5.0~6.8)
Ilan County	0.7 (0.4~1.1)	1.5 (0.8~2.2)	0.7 (0.4~1.0)	0.4 (0.1~0.7)	3.3 (1.8~4.8)
Miaoli County	5.1 (2.5~7.9)	11.3 (5.4~17.6)	3.9 (1.9~5.9)	3.0 (0.7~6.2)	23.3 (11.1~35.0)
Changhua County	5.2 (4.3~6.2)	8.8 (7.4~10.2)	5.2 (4.5~6.0)	2.8 (0.8~4.9)	22.1 (18.8~25.9)
Nantou County	6.1 (3.2~9.2)	12.3 (6.5~18.6)	5.8 (3.0~8.7)	4.5 (1.2~8.9)	28.7 (14.5~43.9)
Yunlin County	5.7 (3.9~7.6)	9.7 (6.8~12.8)	6.1 (4.3~7.8)	2.7 (0.8~4.9)	24.2 (17.0~30.9)
Chiayi County	3.3 (2.7~3.9)	6.1 (5.1~7.1)	3.8 (3.3~4.3)	1.9 (0.7~3.2)	15.1 (13.0~17.4)
Pingtung County	3.8 (2.6~5.2)	7.5 (5.1~10.2)	3.1 (2.1~4.2)	1.9 (0.7~3.4)	16.3 (11.1~21.9)
Hualian County	0.6 (0.4~0.7)	1.7 (1.4~2.1)	0.6 (0.5~0.8)	0.3 (0.1~0.6)	3.2 (2.5~4.0)
Taitung County	0.4 (0.3~0.5)	0.4 (0.3~0.5)	0.2 (0.1~0.2)	0.1 (0.0~0.2)	1.0 (0.8~1.3)
Taiwan	81.7 (75.3~87.9)	154.4 (143.2~166.0)	81.4 (76.4~86.7)	42.0 (32.5~52.0)	359.6 (334.8~384.9)

Abbreviation: IHD: Ischemic Heart Disease, LC: Lung Cancer, COPD: Chronic Obstruct Pulmonary Disease, CI: Confidence Interval; Unit in deaths/year.
