# Supplementary material for: County-Wide Mortality Assessments Attributable to PM2.5 Emissions from Coal Consumption in Taiwan
Source: Int J Environ Res Public Health. 2022 Jan 30;19(3):1599. doi: 10.3390/ijerph19031599 (PMC8835574; doi:10.3390/ijerph19031599)
Supplement: Supplementary file 1 [file ijerph-19-01599-s001.zip › Table S4.pdf]

Table S4. Disease-specific pre-mature deaths attributable to ambient $PM_{2.5}$ in different cities/counties, Taiwan

	IHD	Stroke	LC	COPD	Total Death
City/County	n (95%CI)	n (95%CI)	n (95%CI)	n (95%CI)	n (95%CI)
Taipei City	57.3 (46.8~67.8)	89.1 (78.1~100.5)	50.0 (42.8~56.6)	4.9 (1.8~8.6)	200.9 (182.4~220.9)
Taichung City	62.0 (51.8~72.9)	127.0 (112.5~140.4)	71.1 (62.6~78.9)	7.8 (3.6~12.4)	268.0 (247.8~287.7)
Tainan City	51.8 (43.5~59.3)	119.3 (104.7~132.4)	62.9 (55.4~69.9)	7.0 (2.3~12.6)	241.1 (222.3~258.5)
Kaohsiung City	102.0 (86.0~116.9)	181.5 (162.2~199.1)	110.5 (96.2~122.8)	16.3 (6.8~26.9)	410.3 (378.9~439.4)
Keelung City	9.5 (7.8~11.1)	17.3 (15.1~19.5)	7.6 (6.6~8.5)	1.0 (0.3~1.7)	35.5 (32.5~38.5)
Hsinchu City	10.9 (8.8~12.8)	16.4 (14.0~18.5)	9.1 (8.1~10.1)	0.1 (0.0~0.3)	36.5 (33.1~39.7)
Chiayi City	8.2 (6.8~9.7)	12.9 (11.4~14.3)	8.3 (7.1~9.2)	2.0 (0.8~3.3)	31.3 (28.8~34.0)
New Taipei City	83.2 (68.2~98.5)	150.4 (131.3~167.1)	87.8 (76.6~99.0)	9.6 (3.7~15.7)	331.0 (303.6~358.0)
Taoyuan City	34.4 (28.4~40.5)	90.2 (79.6~100.5)	39.4 (34.6~44.1)	3.4 (1.6~5.3)	167.4 (153.1~180.5)
Hsinchu County	13.1 (10.8~15.3)	32.2 (38.4~36.0)	6.6 (5.6~7.4)	0.5 (0.1~1.0)	52.3 (47.3~57.1)
Ilan County	8.3 (6.7~9.7)	21.8 (19.2~24.4)	8.0 (7.0~8.9)	0.9 (0.2~1.8)	39.0 (35.8~42.4)
Miaoli County	15.3 (12.1~18.4)	30.2 (26.5~33.6)	12.3 (10.8~13.8)	1.2 (0.2~2.3)	59.0 (54.0~64.0)
Changhua County	35.2 (29.1~41.6)	68.0 (59.5~75.9)	32.8 (28.4~37.0)	3.9 (1.3~6.9)	139.9 (127.0~151.3)
Nantou County	17.6 (14.3~20.6)	39.3 (34.7~44.2)	17.3 (14.8~19.4)	2.9 (1.0~5.0)	77.2 (70.8~84.0)
Yunlin County	25.8 (21.4~30.4)	50.2 (44.3~56.0)	27.7 (23.8~31.7)	2.3 (0.7~4.2)	106.0 (96.2~115.9)
Chiayi County	18.3 (15.3~21.1)	28.4 (24.8~31.7)	21.7 (18.8~24.6)	3.6 (1.3~6.4)	72.0 (65.6~78.1)
Pingtung County	31.7 (24.4~39.8)	66.6 (53.3~78.7)	26.0 (20.7~31.6)	6.7 (2.7~11.5)	131.1 (107.0~155.0)
Hualian County	6.7 (5.4~8.0)	26.1 (22.5~29.4)	7.4 (6.4~8.3)	0.9 (0.3~1.6)	41.0 (37.1~44.9)
Taitung County	11.5 (9.4~13.5)	7.8 (6.7~8.9)	2.8 (2.4~3.2)	0.2 (0.0~0.4)	22.3 (20.0~24.7)
Taiwan	602.6 (571.3~632.6)	1174.8 (1134.0~1214.6)	609.0 (584.3~633.3)	75.4 (59.4~91.8)	2461.7 (2401.1~2529.1)

Abbreviation: IHD: Ischemic Heart Disease, LC: Lung Cancer, COPD: Chronic Obstruct Pulmonary Disease, CI: Confidence Interval; Unit in deaths/year.
